# Global phenotypic characterisation of human platelet lysate expanded MSCs by high-throughput flow cytometry

**DOI:** 10.1038/s41598-018-22326-5

**Published:** 2018-03-02

**Authors:** Monica Reis, David McDonald, Lindsay Nicholson, Kathrin Godthardt, Sebastian Knobel, Anne M. Dickinson, Andrew Filby, Xiao-nong Wang

**Affiliations:** 1 Haematological Sciences, Institute of Cellular Medicine, Newcastle upon Tyne, United Kingdom; 2 Flow Cytometry Core Facility, Faculty of Medical Sciences, Newcastle University, Newcastle upon Tyne, UK; 3 Miltenyi Biotec GmbH, Bergisch Gladbach, Germany

## Abstract

Mesenchymal stromal cells (MSCs) are a promising cell source to develop cell therapy for many diseases. Human platelet lysate (PLT) is increasingly used as an alternative to foetal calf serum (FCS) for clinical-scale MSC production. To date, the global surface protein expression of PLT-expended MSCs (MSC-PLT) is not known. To investigate this, paired MSC-PLT and MSC-FCS were analysed in parallel using high-throughput flow cytometry for the expression of 356 cell surface proteins. MSC-PLT showed differential surface protein expression compared to their MSC-FCS counterpart. Higher percentage of positive cells was observed in MSC-PLT for 48 surface proteins, of which 13 were significantly enriched on MSC-PLT. This finding was validated using multiparameter flow cytometry and further confirmed by quantitative staining intensity analysis. The enriched surface proteins are relevant to increased proliferation and migration capacity, as well as enhanced chondrogenic and osteogenic differentiation properties. *In silico* network analysis revealed that these enriched surface proteins are involved in three distinct networks that are associated with inflammatory responses, carbohydrate metabolism and cellular motility. This is the first study reporting differential cell surface protein expression between MSC-PLT and MSC-FSC. Further studies are required to uncover the impact of those enriched proteins on biological functions of MSC-PLT.

## Introduction

Mesenchymal stem cells (MSCs) are multipotent progenitor cells bearing a wide variety of biological activities including mesodermal differentiation potential, stromal support and immunomodulation^1^. MSCs have shown promising therapeutic potential for a wide variety of medical conditions^2,3^. Due to their low frequency *ex vivo* expansion is a pre-requisite to achieve clinically relevant cell numbers. Conventional expansion of MSCs relies on the use of foetal calf serum (FCS) as a culture supplement, which provides essential nutrients and growth factors required to support cell growth. Concerns have been raised by regulatory authorities regarding the use of FCS-containing culture media to expand MSCs for clinical use as it is a potential source of unidentified zoonoses and prions that could prompt xenogeneic infections^4–7^. FCS has also been shown to have high lot-to-lot variability and to elicit immune responses in patients who have received repeated administrations of FCS-expanded MSCs^6,7^. To eliminate potential risks associated with the use of FCS, intensive research has been carried out to identify a suitable replacement of human origin. Previous findings have demonstrated the superiority of human platelet lysate (PLT) over human AB serum^8^, human plasma^9^ and human autologous serum^10^ for *ex vivo* expansion of MSCs, owing to its high content of growth factors, low cost and ease in large-scale production^11–14^. This present study focuses on validating the concept and potential benefit of replacing FCS with PLT in MSC production for clinical use rather than comparing PLT with other human blood products. PLT is a concentrated solution of thrombocyte-derived growth factors produced by mechanical disruption of activated platelets using freeze-thaw procedures^15^. Currently, industrial scale production of GMP grade PLT utilises the pooling of platelet units derived from a large number of donors, resulting in a product with low lot-to-lot variability, as well as high consistency and purity^16^. The use of PLT in MSC expansion has been shown to promote proliferation while maintaining multi-lineage differentiation potential and immunomodulatory properties^11,12,15,17^. In the past decade, MSCs expanded in PLT-containing media have been increasingly used in clinical trials^18,19^. Despite encouraging clinical outcome, the global surface protein expression of PLT-expanded MSC is yet to be uncovered.

Given the importance of cell surface proteins to a plethora of biological functions this study characterised the phenotypic profile of MSCs expanded in PLT-containing media (MSC-PLT), in comparison to conventional FCS-expanded MSCs (MSC-FCS), using high-throughput flow cytometry. This analysis platform is a reproducible discovery-orientated screening technology that allows for the identification of surface proteins using panels of hundreds of CD markers and enables the use of multiplexed assays for the analysis of several cell populations simultaneously^20–22^. It takes advantage of the general characteristics of conventional flow cytometry for cell surface protein expression analysis but incorporates a high-speed sample loading device that allows for fast analysis of a range of antibodies, usually arrayed in 96- or 384-well plates^20–22^. This tool can also be combined with fluorescent cell barcoding that allows for parallel analysis of distinct populations^20–22^. To date, no high-throughput flow cytometry analysis has been reported on MSC-PLT, although such analyses have been used to screen surface proteins of MSC-FCS and other cell types^21,23–27^. This study characterises the overall phenotypic ‘finger print’ of MSC-PLT to identify differentially expressed surface proteins compared to MSC-FCS. This study also provides a searchable database of cell surface proteins expressed by MSCs expanded under different culture conditions that can be used as a resource to appreciate the heterogeneity and functional diversity of the MSC lineage.

## Results

### Characterisation of MSC-FCS and MSC-PLT

**Figure 1:**
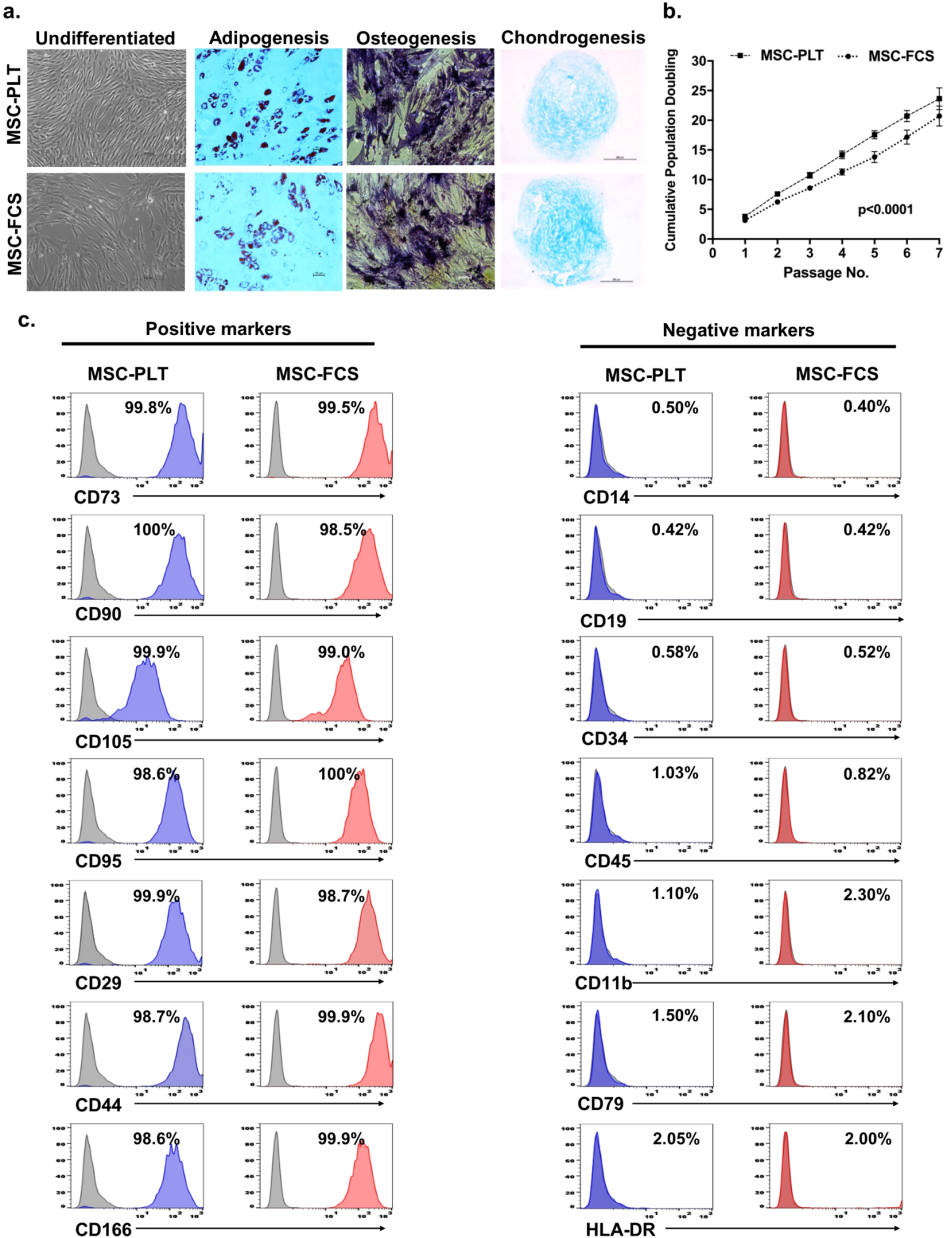
Characteristics of MSC-PLT and MSC-FCS. (**a**) Representative images of MSC-PLT and MSC-FCS showing their morphology and ability to differentiate into adipocytes, osteoblasts and chondroblasts; Scale bars represent 100 or 200 μm; (**b**) Cumulative population doublings of MSC-PLT and MSC-FCS presented as the mean ± SEM of 13 independent samples; (**c**) Representative histograms of MSC-PLT and MSC-FCS phenotype showing comparable expression profile of the positive and negative markers.

Human bone marrow derived MSCs from healthy donors were expanded in parallel in culture media containing either 5% PLT or 10% FCS. At passage 3, MSCs were characterised based on morphology, tri-lineage differentiation potential and phenotype as described by the ISCT criteria^28^. MSC-PLT and MSC-FCS exhibited comparable spindle-shaped morphology (Fig. 1a). The ability to differentiate into adipocytes, osteoblasts and chondroblasts was observed in both MSC populations as examined by Oil-Red-O, alkaline phosphatase/Von Kossa and Alcian blue staining, respectively (Fig. 1a). Analysis of cumulative population doublings of paired samples showed that MSC-PLT had higher proliferative capacity than MSC-FCS (p < 0.0001) (Fig. 1b). The paired MSC samples also exhibited the characteristic phenotype, positive for the expression of CD73, CD90, CD105, CD95, CD29, CD44 and CD166 whilst negative for the expression of CD14, CD19, CD34, CD45, CD11b, CD79 and HLA-DR (Fig. 1c).

### MSC-PLT show a differential surface protein expression pattern compared to MSC-FCS

**Figure 2:**
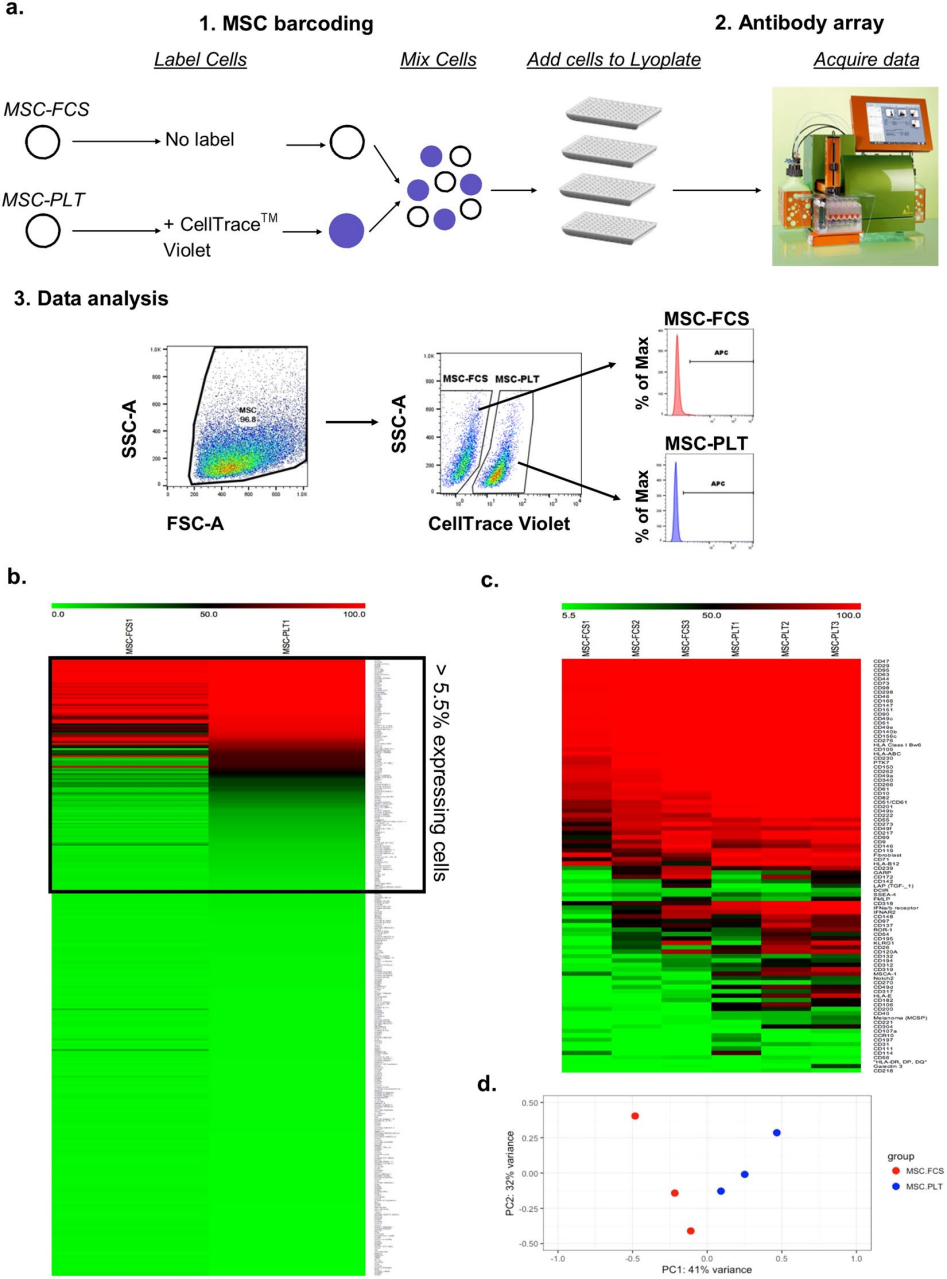
MSC-PLT show a differential surface protein expression pattern compared to MSC-FCS. (**a**) Experimental overview of MSC surface protein screening using the Lyoplate technology. MSC-PLT and MSC-FCS were discriminated by labelling MSC-PLT with celltrace violet. Both samples were then mixed and seeded into wells of 96-well plates containing an array of APC-conjugated antibodies. Data was acquired using the automated MACSQuant flow cytometer and analysed by manual gating using MACSQuantify or FlowJo software. MSCs were selected based on SSC/FSC properties. MSC-FCS and MSC-PLT were gated as Celltrace^TM^ violet negative and positive populations, respectively, and the percentage of positive cells for each antibody was determined based on gates drawn for each isotype control. (**b**) Heatmap showing the expression of all 356 markers on the surface of MSC-FCS and MSC-PLT. (**c**) Heatmap depicting the expression of the 99 markers detected as positive on the surface of MSC-FCS and MSC-PLT samples. (**d**) Principle component analysis plot illustrating the differences between MSC-PLT and MSC-FCS sample cohorts.

The surface protein expression of both MSC populations was analysed using the Lyoplate system consisting of a panel of monoclonal antibodies specific for 356 human surface proteins. A general overview of the experimental design is illustrated in Fig. 2a. Briefly, paired MSC samples at passage 3 were used for surface protein analysis. To discriminate between MSC-PLT and MSC-FCS, MSC-PLT populations were labelled with CellTrace^TM^ Violet, while MSC-FCS populations were kept unlabelled. MSC-FCS and labelled MSC-PLT cells were then pooled and arrayed on 96 well plates containing APC-conjugated antibodies and isotype controls. APC positivity for each specific antibody in MSC-PLT and MSC-FCS was determined as illustrated in the gating strategy in Fig. 2a.

An initial surface protein screening was performed on one paired MSC-PLT and MSC-FCS samples to identify the positively expressed proteins, which were defined by showing positive staining in at least 5.5% of the cells in one or both cell populations. This threshold has been used as standard criteria in similar studies^23–25^. Overall, 99 out of 356 markers were positively expressed on both cell populations while the remaining markers were neither expressed on MSC-PLT nor MSC-FCS (Fig. 2b). Both MSC populations expressed the characteristic markers, positive for CD73, CD90, CD105, CD44 and CD29 and negative for CD45, CD34, CD3, CD19, CD14, CD11b and CD79, suggesting the reliability of this analysis platform. Detailed information on the 356 analysed proteins and their expression on MSC-PLT and MSC-FCS is presented in Supplementary Table S1.

The 99 surface proteins positively expressed on both MSC populations were further analysed in three paired MSC samples generated from three independent bone marrow donors. The results showed that 51 out of 99 positively expressed proteins were equally highly expressed on both MSC populations, including those that have been consistently detected in previous studies, such as, CD46, CD47, CD49a, CD9, CD81, CD63, CD47, among others^23–25,29^. The remaining 48 proteins displayed variable expression levels in MSC-PLT and MSC-FCS (Fig. 2c). Principal component analysis (PCA) was performed to illustrate the differences in global surface protein expression patterns between the two MSC populations. PCA analysis segregated MSC-PLT and MSC-FCS into two distinct clusters. In addition, a lower variability was depicted within the MSC-PLT samples, with all samples closely clustered together, whilst MSC-FCS samples were spread across the spectrum, suggesting that MSC-PLT could be less heterogeneous compared to their MSC-FCS counterpart (Fig. 2d). The percentage of cells expressing the positively detected surface proteins in MSC-PLT and MSC-FCS is presented in Supplementary Table S2, which provides a searchable database for future studies.

### Thirteen surface proteins are significantly enriched on MSC-PLT

**Figure 3:**
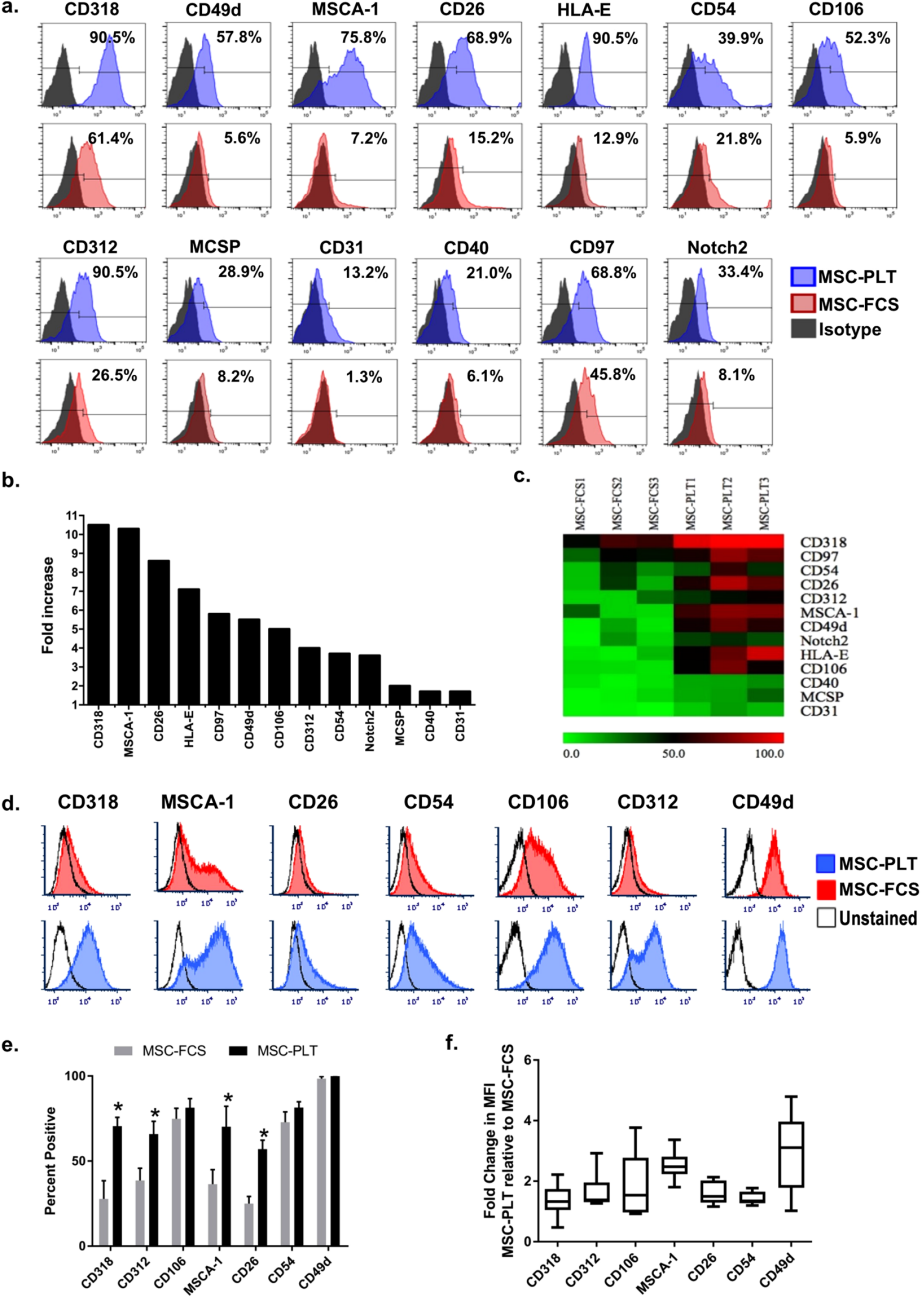
Surface proteins significantly enriched in MSC-PLT. From the analysis of the 48 surface proteins showing variable expression between MSC-PLT and MSC-FCS, 13 of which were significantly enriched on MSC-PLT. (**a**) Representative histograms of the 13 enriched surface proteins in MSC-PLT (blue) and MSC-FCS (red) as assessed by the Lyoplate analysis. (**b**) Average fold increase in percentage of positive cells in MSC-PLT (n = 3). (**c**) heatmap depicting the hierarchical clustering of the 13 markers significantly upregulated in MSC-PLT. (**d**) Representative histograms of enriched surface proteins in MSC-PLT compared to MSC-FCS in a validation cohort (n = 6), using multiparameter flow cytometry. Surface protein expression was evaluated by (**e**) the percentage of positive cells in the gated live cell single cell population; data represents mean ± SEM and *p < 0.05 and (**f**) the fold change of median fluorescence intensity of positively gated cells (MSC-PLT relative to MSC-FCS).

To identify the differentially expressed surface proteins we focused on the 48 proteins that had a variable expression in MSC-PLT and MSC-FCS. All 48 proteins showed a higher percentage of positive cells in MSC-PLT compared to MSC-FCS, of which 13 were significantly enriched on MSC-PLT with a fold increase in percentage of positive cells of ≥1.5 and a p-value < 0.05 (Fig. 3a,b). The unsupervised hierarchical clustering using percentage value of positive cells of these 13 proteins stratified MSC-PLT and MSC-FCS into two distinct clusters (Fig. 3c). No downregulation of surface proteins was detected in MSC-PLT compared to MSC-FCS in our study (Supplementary Table S2). None was reported in the literature either.

To validate the differentially expressed surface proteins detected using high-throughput screening, an independent validation cohort of paired MSC-PLT and MSC-FCS samples were collected (n = 6) and analysed for the expression of the surface proteins that were highly enriched on MSC-PLT, namely CD318, MSCA-1, CD26, CD54, CD106, CD312 and CD49d, using multiparameter flow cytometry. In comparison with MSC-FCS, higher percentage of positive cells was detected in MSC-PLT for all analysed surface proteins, with the difference in CD318, CD312, MSCA-1 and CD26 reached statistical significance (Fig. 3d,e). Further analysis of MFI for each surface protein confirmed the finding of Lyopate analysis, with MSC-PLT exhibiting higher expression of all analysed antigens across all paired MSC samples (Fig. 3f).

To explore the molecular mechanisms underpinning the differential surface protein expression between MSC-PLT and MSC-FCS, qRT-PCR was performed in an independent cohort of paired MSC samples (n = 3) to quantify the mRNA expression levels of CD318 *(CDCP1)*, MSCA-1 (*ALPL*), and CD26 (*DPP4*), which are the top three most significantly enriched surface proteins expressed on MSC-PLT with the highest fold increase in the percentage of positive cells (Fig. 3b). The analysis revealed that MSCA-1 (*ALPL*) and CD26 (*DPP4)* were significantly overexpressed in MSC-PLT at mRNA levels (p < 0.05). MSC-PLT also exhibited a clear trend of elevated mRNA level for CD318 (*CDCP*) (Fig. S1).

### Quantitative analysis of the enriched surface proteins on MSC-PLT

**Figure 4:**
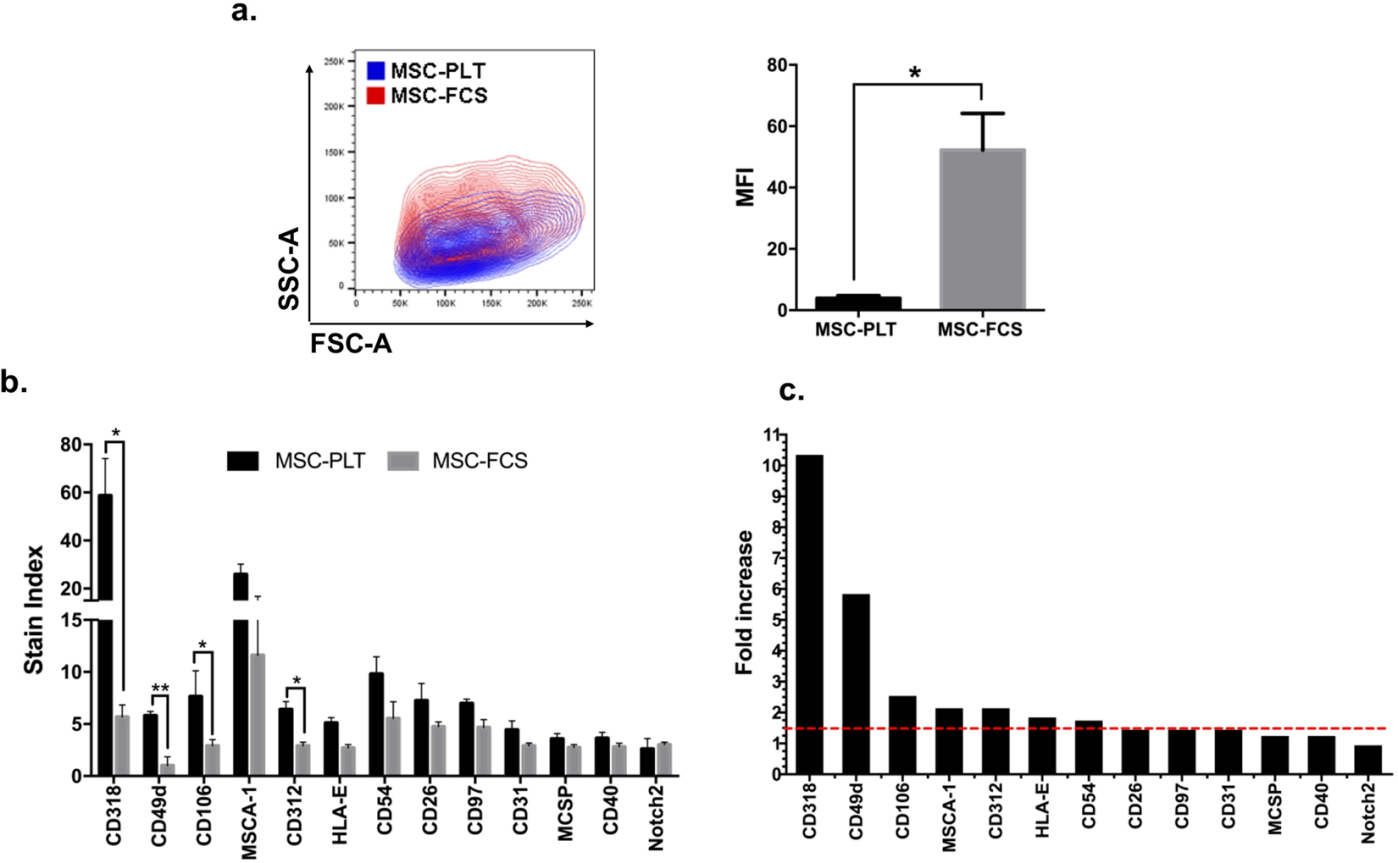
Stain index of enriched surface proteins in MSC-PLT. (**a**) Representative contour plot of paired MSC samples with MSC-PLT displaying smaller size and lower complexity than MSC-FCS. MSC-FCS showed significantly higher background fluorescence, herein defines as median fluorescence intensity (MFI), in the APC channel, than MSC-PLT. (**b**) Stain index of 13 surface proteins enriched on MSC-PLT, in comparison with MSC-FCS (n = 3); error bars represent mean ± SEM and *p-value < 0.05. (**c**) Fold increase in stain index shown as MSC-PLT relative to MSC-FCS). Red line represents a fold increase of 1.5.

To quantify the expression levels of the enriched surface proteins identified in MSC-PLT, we analysed the differences in staining intensity, herein expressed as stain index (SI), between MSC-PLT and MSC-FCS. The SI is a measure that allows the comparison of the relative brightness of a fluorochrome. The SI takes into consideration of the background fluorescence which can be influenced by autofluorescence, nonspecific staining, electronic noise and optical background from other fluorochromes^30^. As illustrated in Fig. 4a, MSC-FCS displayed higher complexity than MSC-PLT, as evidenced by increased SSC-A properties. This complexity further influences the background fluorescence of MSC-FCS, which displayed a 13-fold higher background fluorescence intensity than MSC-PLT (p < 0.05). Despite the higher background intensity of MSC-FCS, higher SI was detected in MSC-PLT for 10 out of 13 proteins. The proteins CD318 and CD49d were the most enriched proteins in MSC-PLT exhibiting 10.4-fold and 5.9-fold higher SI than MSC-FCS respectively (p < 0.05 for both). Significantly higher expression of the surface protein CD106 was also detected in MSC-PLT, showing a 2.6-fold higher SI than MSC-FCS (p < 0.05). The surface proteins MSCA-1, CD312, HLA-E, CD54, CD26, CD97 and CD31 showed concomitant ≥1.5-fold higher SI in MSC-PLT (ranging from 2.2-fold to 1.5-fold). However, a statistical significance was not reached in the small sample cohort tested (n = 3). The remaining 3 proteins, MCSP, CD40, and Notch2 showed comparable SI levels between MSC-PLT and MSC-FCS (Fig. 4b,c). A summary of median fluorescence intensity and SI values for all positively expressed markers is presented in Supplementary Table S3, which provides a searchable database for future studies.

### Pathway and Network enrichment analysis

**Figure 5:**
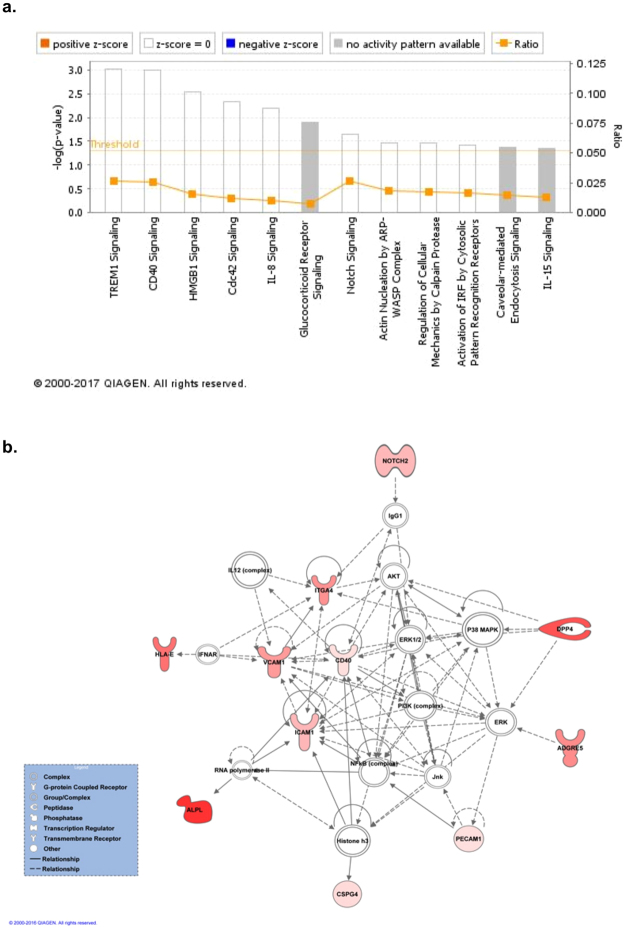
Canonical pathway and network analysis by IPA. The 13 enriched proteins in MSC-PLT were analysed through the use of Ingenuity Pathway Analysis (IPA, QIAGEN INC., https://www.qiagenbioinformatics.com/products/ingenuity-pathway-analysis). (**a**) Significantly altered canonical pathways associated with the enriched surface proteins in MSC-PLT. The canonical pathways included in this analysis are shown in the x-axis of the bar chart. The y-axis indicates the statistical significance calculated using the right-tailed Fisher exact test. (**b**) Graphical representation of the top ranked network, related to inflammatory responses, with a score of 28, as nodes (molecules) and edges (biological relationship between the different molecules). Red nodes represent the investigated enriched proteins involved in this network. Meanings of node shapes and edges are indicated in the legend within the figure.

The 13 enriched proteins in MSC-PLT were analysed through the use of Ingenuity Pathway Analysis (IPA, QIAGEN INC., https://www.qiagenbioinformatics.com/products/ingenuity-pathway-analysis) to identify enriched pathways and associated networks based on a calculated probability score of ≥2 and a p-value of <0.05 using IPA software application. The top statistically significant (p < 0.05) canonical pathways influenced by these 13 proteins were shown in Fig. 5a. The pathways associated with “TREM1 signalling”, “CD40 signalling” and “HMGB1 signalling” were the most significantly enriched pathways in MSC-PLT (p < 0.001 for all). These pathways have been previously reported to be involved in the regulation of inflammatory responses and osteogenic differentiation of MSCs^31–33^.

From the data for canonical pathways and biological functions, IPA assessed networks which revealed the direct or indirect association of the 13 enriched markers in MSC-PLT with each other, and other molecules identified in the canonical pathways. The IPA software identified 3 different networks. The first and most relevant network is depicted in Fig. 5b. This network has a score of 28, included 11 of 13 the analysed markers and is associated with inflammatory responses. This network’s key components included p38 mitogen-activated protein kinases (MAPK), extracellular signal-regulated kinase (ERK) 1/2 and protein kinase B/phosphatidylinositol 3-kinase (AKT/PI3K) family of kinases. The scores and number of markers associated to the second and third networks are depicted in Table 1. The second network, scoring 9, is associated with the carbohydrate metabolism, and contains 4 of the 13 analysed proteins. This network has as key component the G-protein-coupled receptor (Gpcr) family, which mediates the energy metabolism and lead to the activation of important pathways such as the vascular endothelial growth factor (VEGF)^34^ and the insulin growth factor (IGF) pathway^35^. The third network, with a score of 4, is associated to the cellular mobility and migration and includes 2 of the 13 enriched markers. This network has as central component the GTPase family which regulate the actin cytoskeleton and the mobility of various cells^36^. Details of the components of each network are represented in Supplementary Table S4.

**Table 1:** Summary of biological networks derived using IPA network analysis, with respective scores, number of analysed proteins and key components to each specific network.

**Networks**	**Score**	**Number analysed proteins in network**	**Key component (s)**
Inflammatory response	28	11	ERK1/2; P38 MAPK; AKP/PI3K complex
Carbohydrate metabolism	9	4	Gpcr
Cellular movement	4	2	GTPase

## Discussion

Human PLT has been shown, as a xenogeneic-free supplement for MSC expansion, to promote the proliferation and genomic stability of MSCs while maintaining their multipotent properties. MSCs expanded in PLT-containing media have been increasingly used in clinical trials. Yet the global surface protein expression of MSC-PLT remains unreported. Here, we present novel data demonstrating the expression pattern of 356 surface proteins on MSC-PLT screened using discovery-orientated high-throughput flow cytometry. This screening platform has been successfully used for the identification of surface markers in several alternative cell types^21,24,37,38^.

Among 99 surface proteins that were positively expressed on both MSC-PLT and MSC-FCS, 48 were enriched on MSC-PLT compared to MSC-FCS. Further principal component analysis of the overall expression profiles revealed a lower variability within the MSC-PLT sample cohort compared to the MSC-FCS cohort. Previous reports have shown that PLT expanded MSCs exhibited smaller and narrower morphology than FCS-expanded MSCs^17,39^. This morphology was further associated to lower contamination with other resting mature cell types and higher uniformity of the cell surface protein expression, suggesting that PLT expanded MSCs consist of a more homogeneous population than their FCS-expanded counterparts^40^. Consistent with the previous findings, the enriched expression of the 48 variably expressed proteins on MSC-PLT detected in our study suggests an increased homogeneity of PLT expanded MSCs.

Beyond serving as markers for cell characterisation, surface proteins are critical mediators of important biological functions including cell-to-cell contact, extracellular matrix interactions, cell migration and signal transduction. The main objective of our study was to address whether there are differences in the surface protein expression profiles between MSC-PLT and MSC-FCS. We identified 13 surface proteins (CD40, HLA-E, MSCA-1, CD97, CD106, CD54, Notch2, CD318, CD26, CD312, MCSP, CD31 and CD49d) that were significantly enriched on MSC-PLT. Several of the overexpressed markers have been shown to be heterogeneously expressed by FCS-expanded MSCs^41–46^. In comparison to FCS, human PLT contains high concentration of growth factors including fibroblast growth factor (FGF), epidermal growth factor (EGF), transforming growth factor (TGF-β1), platelet derived growth factor (PDGF), insulin growth factor-1 (IGF-1), and vascular endothelial growth factor (VEGF)^12^. Collectively, these growth factors are involved in repair mechanisms by the activation of signalling pathways involved in cell proliferation, recruitment and motility thus promoting wound healing and neovascularisation^47–49^, therefore, they may have a direct effect on the increased expression of the 13 identified surface proteins.

In our study, the proteins CD318, CD26 and MSCA-1 showed the highest enrichment in MSC-PLT, which may have contributed to the enhanced proliferative capacity of PLT expanded cells. CD318, also known as CUB-domain-containing protein 1 is a type I transmembrane protein involved in cell adhesion and extracellular matrix interaction^43^. CD318 is expressed mainly by cultured MSCs^50^ and confers higher proliferative capacity to adherent progenitors^43^. CD26 is a surface glycoprotein with peptidase activity that has been detected on the surface of various cells^51^. It has been demonstrated that the expression of CD26 on MSCs was associated with higher CFU-F properties^52^. MSCA-1, also known as alkaline phosphatase liver/bone/kidney, has been associated with a subpopulation of MSCs with enhanced chondrogenic and osteogenic differentiation properties^41,50,53^.

Other enriched proteins, including CD97, CD312, MCSP and Notch2, have been reported to be expressed on the surface of MSCs but their relevance is still being investigated^54–56^. These proteins may have an important role in MSC proliferation and adhesion. CD97 and CD312, two closely related members of the EGF-TM7 transmembrane receptors, are important mediators of stemness and invasiveness in several carcinomas^57^. Similarly, MCSP, a transmembrane receptor with high affinity to a range of integrins and growth factors, including, bFGF and PDGF-AA, has been shown to promote cellular migration and metastasis^58^, while Notch2 signalling regulates cell proliferation^56^. Some of the 13 proteins enriched on MSC-PLT belong to the family of cell adhesion molecules, including CD49d, CD31, CD54 and CD106. The expression of CD49d has been detected in MSCs in a previous study^59^; however, the biological relevance of CD49d in MSCs has not yet been elucidated. CD31 is commonly regarded as a negative marker in MSCs. In our study, a small percentage of MSC-PLT (13.8% ± 2.3 SEM) had positive CD31 expression whilst MSC-FCS showed negativity for this marker (2.0% ± 0.6 SEM). The expression of CD31 has been shown to be up-regulated by PDGF, FGF and other growth factors^60,61^, therefore its enrichment on MSC-PLT may be a result of the high concentration of these growth factors in the PLT. Expression of the adhesion molecules CD54 and CD106 has been reported to identify a subpopulation of MSCs with enhanced immunosuppressive properties^42,62^.

Lack of expression in co-stimulatory molecules CD80, CD86 and CD40 is generally considered as a feature of MSCs that is associated to their low immunogenic properties^63^. In our study, there was a small proportion of MSC-PLT that expressed very low levels of CD40 (18.3% ± 1.4 SEM) while MSC-FCS were detected negative (4.9% ± 2.0 SEM) for this antigen. This marker binds to its ligand CD40L and contributes to the pro-inflammatory signalling cascade^32^. This up-regulation of pro-inflammatory markers may indicate an impaired immunogenic potential of MSC-PLT in comparison to their MSC-FCS counterparts. However, further investigation is necessary to evaluate whether this enrichment is biologically relevant in MSC-PLT immunogenicity. MSC-PLT also showed enhanced expression of HLA-E. This protein is a non-classical major histocompatibility complex (MHC) which binds to CD94/NKG2A receptor in NK cells and activates a signalling cascade which inhibits NK cell cytotoxicity^64^. Increased expression of HLA-E by MSC-PLT indicates these cells may be less susceptible to NK cell mediated rejection. In fact, one previous report illustrated that PLT-expanded MSCs show increased resistance to NK cell mediated lysis^29^.

*In silico* network analysis with the 13 upregulated markers, showed a significant enrichment of inflammatory responses, carbohydrate metabolism and cell movement pathways. The most significant network, related to inflammatory responses, includes the protein complexes, ERK, PI3K and p38 MAPK^65,66^, which are essential signalling cascades involved in cellular growth and differentiation. This pathway is an important regulator of both pro- and anti-inflammatory responses^67^. Activation of these pathways is mediated by a variety of growth factors, including FGF, VEGF and TGF-β1^68^, all of which are detected at high concentration in platelet lysate product. The carbohydrate metabolism pathway, having as key components the Gpcr family, is involved in regulation of cell metabolism^69^ and implicated in their proliferative and migratory capacity^70^. The cell movement pathway has as key components the Rho family of guanosine triphosphatases (GTPases), which play important roles in cytoskeletal rearrangements that are crucial in a set of specialised functions, including, self-renewal, adhesion and migration^36^. In MSCs, the Rho-GTPase signalling regulates commitment into osteogenic and adipogenic differentiation^71^.

In conclusion, this is the first study reporting a differential cell surface protein expression between MSC-PLT and MSC-FCS. The identified cell surface proteins enriched in MSC-PLT are potentially associated with increased proliferation and migration capacity, as well as enhanced chondrogenic and osteogenic differentiation properties. Further studies are required to further investigate into the activation status of the identified pathways in MSC-PLT and uncover the impact of those enriched proteins on biological functions of MSC-PLT. This study also provides a searchable database of cell surface proteins expressed by MSCs expanded in different culture media, which can inform further studies to characterise specific MSC subsets.

## Materials and Methods

### Human bone marrow-derived MSCs

Each MSC sample was derived from independent bone marrow aspirates collected from different healthy donors (surplus of haematopoietic stem cell transplant) with informed consent and Local Research Ethical Committee approval (NRES Committee North East – Newcastle & North Tyneside 2). All methods were performed in accordance with the relevant guidelines and regulations. All MSC samples were derived from bone marrow mononuclear cells using standard plastic adherent method. Each paired MSC-PLT and MSC-FCS sample was generated from the same bone marrow sample by splitting the bone marrow mononuclear cells into two sub-fractions, one was cultured in DMEM based non-haematopoietic media (Miltenyibiotec, Germany) supplemented with 10% FCS while the other in DMEM (Sigma-Aldrich, USA) supplemented with 5% human platelet lysate (PLTmax, Mill Creek, USA) and 2 IU/ml Heparin. The same FCS and PLT products were used across all cultures throughout the study. The platelet lysate utilised in this work was commercially available and manufactured under good manufacturing practice (GMP) conditions. The specifications about the composition and manufacturing process of the PLT used in this study are described in details by Crespo-Diaz R *et al*.^12^ Both media were also supplemented with 2 mM L-glutamine, 100 IU/ml penicillin and 100 µg/ml streptomycin (Sigma-Aldrich, USA). Paired MSC samples were then incubated at 37 °C, 5% CO_2_ humidified atmosphere. Non-adherent cells were removed after 48 h by media changes. The adherent cells were maintained in culture, with media changed twice weekly. Cells were then passaged when reaching 70–80% confluence using 1x Trypsin-EDTA (Sigma-Aldrich, USA) and plated at a density of 4 × 10^3^ cells/cm^2^. Cells harvested from passage 3 were used for all subsequent analysis. Phenotype, morphology, differentiation capacity and proliferation kinetics was monitored on paired samples as described in supplementary methods.

### Cell barcoding for discrimination of MSC populations

To discriminate between MSC-FCS and MSC-PLT, MSC-PLT were labelled with CellTrace^TM^ Violet (Life Technologies, USA), and MSC-FCS were kept unlabelled. MSC-FCS were suspended in PEB buffer, consisting of phosphate buffered saline (PBS) with 2 mM of EDTA (Sigma-Aldrich, USA), and 0.5% of bovine serum albumin (Sigma-Aldrich, USA), and used directly for subsequent analyses. MSC-PLTs were washed in PBS and re-suspended in a 0.2 µM solution of CellTrace^TM^ violet at a density of 1 × 10^4^ cells/µl. Cells were incubated for 5 minutes at room temperature in the dark, washed twice with PEB and re-suspended in the same buffer for subsequent analyses.

### Surface protein screening using Lyoplate technology

Non-labelled MSC-FCS and Celltrace^TM^ violet labelled MSC-PLT were pooled at a 1:1 ratio and analysed using the Miltenyi 96 well human antibody panel (Miltenyibiotec, Germany), containing APC-conjugated antibodies with specificity for 356 cell surface markers and 9 isotype controls, arrayed on four round bottomed 96-well plates. A detailed list of the antibodies can be found in Supplementary Table S5. Staining and plate preparation was performed as described by the manufacturer’s protocol. Briefly, the lyophilized antibodies were reconstituted with 25 µl of deionized water. The previously prepared MSCs were added to each well of the 96 well plates at a density of 5 × 10^4^ cells/well. Cell incubation with the antibodies was carried out for 10 minutes in the dark at 4 °C. The cells were then washed twice with PEB and centrifuged at 300 × g for 5 minutes. The cell pellet was re-suspended in 50 µl PEB and surface protein expression data was acquired by flow cytometry using an automated MACSQuant flow cytometer (Miltenyibiotec, Germany), where 35 µl of sample were analysed and at least 10,000 events per well were collected. The workflow is shown in Fig. 2a.

### Surface protein screening data analysis

Data acquisition was accomplished using the MACSQuant analyser 10 software (Miltenyibiotec, Germany). Data analysis was performed using either MACSQuant analyser software or FlowJo v10 software (FlowJo LLC, Treestar, USA). An initial analysis template was created to discriminate between MSC-PLT and MSC-FCS and to analyse each population for its APC positivity. Analytical data (event count, percentage of APC positive clean events, median fluorescence intensity and standard deviation) were exported to Excel and associated to sample ID, plate number, row and column. The gating strategy for the flow cytometry data analyses is depicted in Fig. 2a.

### Surface protein validation using multiparameter flow cytometry analysis

To validate the findings of the high throughput screen, paired MSC-PLT and MSC-FCS samples were incubated with an optimally titrated master-mix of antibodies (CD318-FITC, CD312-APC, CD26 PE, CD54 PE-Vio770, MSCA-1 BUV395, CD106 APC-Vio770) or singularly with CD49d-APC. Samples were washed and incubated with DAPI for dead cell exclusion and acquired on a BD FACSymphony A5 flow cytometer (Becton Dickinson, USA) using PMT voltages that were optimised to achieve best resolution without saturating signals in any channel. Detailed Ab information and the detector channels used, including optical filters, are shown in Supplemental Table S6. Spectral compensation was performed using the BD FACSDIVA auto-compensation wizard in conjunction with BD CompBeads (Becton Dickinson) stained with each individual antibody-fluorochrome in the panel. Data was then analysed offline with FCSExpress V6 (DeNovo software, USA) using a classical sequential gating strategy. Briefly, live cells were identified as having an intermediate to high FSC_A value in conjunction with DAPI negativity. Doublets and aggregates were then eliminated using a combination of FSC_A, FSC_H and SSC-W. The percentage of cells (MSC-PLT or MSC-FCS) positive for a given marker was determined relative to their own respective unstained control by setting a gate on the latter to a 99.9% confidence. Median fluorescent intensities were then derived for cells deemed positive for each respective marker only

### Reverse transcription and quantitative Real Time-Polymerase Chain Reaction

RNA from paired MSC samples was extracted using the RNeasy mini kit (Qiagen, Germany) as per the manufacturer’s protocol. RNA purity and quantity was analysed using Nanodrop 2000 (Thermo Fisher Scientific, USA) and reverse transcribed using High-Capacity cDNA reverse transcription reagents (Applied Biosystems, USA). Taqman® (Thermo Fisher Scientific, USA) gene expression assays were used to measure the expression levels of the genes *CDCP1* (CD318, Hs01080405_m1), *ALPL* (MSCA-1, Hs01029144_m1) and *DPP4* (CD26, Hs00897391_m1) all purchased from Thermo Fisher Scientific, USA. Reactions were carried out in triplicate using 7900HT Fast Real Time PCR system (Applied Biosystems, USA) and standard thermal cycling conditions. Ct values were normalised to the reference gene Glyceraldehyde 3-phosphate dehydrogenase (*GAPDH*, Hs9999905_m1, Thermo Fisher Scientific, USA).

### Pathway and network analysis

For further understanding the relevance of the proteins significantly enriched on the surface of MSC-PLT, the 13 enriched markers were analysed through the use of Ingenuity Pathway Analysis (IPA, QIAGEN INC., https://www.qiagenbioinformatics.com/products/ingenuity-pathway-analysis) Canonical pathway analysis identified the pathways from the IPA library of canonical pathway that were most significant to the data set. The markers from the data set that met the fold-change cut-off of 2 in association with a canonical pathway in Ingenuity knowledge base were considered for analysis. The significance of the association between the data set and the canonical pathways was analysed by IPA software algorithms in 2 ways: a ratio of the number of markers from the data set that map to the pathway, divided by the total number of molecules mapping to the canonical pathway, a Fisher exact test was used to calculate a p-value determining the association probability between the molecules in the data set and the canonical pathway. Network analysis was used to exhibit relationships between enriched genes and the canonical pathways identified by the IPA software. Networks were algorithmically constructed by IPA software on the bases of the functional and biological connectivity of the 13 enriched surface proteins on MSC-PLT and by limiting the number of molecules in a network to 35 and displaying only the most significant interactions.

### Statistical analyses

Statistical analyses were performed using GraphPad prism v5.0 (GraphPad software, USA). Unless otherwise stated, data are presented as mean and standard error of the mean (SEM). One-way ANOVAs were used for statistical analysis and p-values of <0.05 were considered significant.

## Electronic supplementary material

Supplementary information

Supplementary Table S1

Supplementary Table S2

Supplementary Table S3

Supplementary Table S5
