# Supplementary material for: Global phenotypic characterisation of human platelet lysate expanded MSCs by high-throughput flow cytometry
Source: Sci Rep. 2018 Mar 2;8:3907. doi: 10.1038/s41598-018-22326-5 (PMC5834600; doi:10.1038/s41598-018-22326-5)
Supplement: Supplementary file 1 — Supplementary information [file 41598_2018_22326_MOESM1_ESM.doc]

**Global phenotypic characterisation of human platelet lysate expanded MSCs by high-throughput flow cytometry**

Monica Reis, David McDonald, Lindsay Nicholson, Kathrin Godthardt, Sebastian Knobel, Anne Dickinson, Andrew Filby, Xiao-nong Wang

**Proliferation kinetics**

Proliferative potential of MSC-PLT was assessed by comparing their growth rate with FCS expanded MSCs. Growth was assessed after passage 1 by measuring the population doubling, i.e., the number of times the cells have doubled in number since their primary isolation. Cumulative population doubling (CPD) was calculated using the following equation: [Log(H)-Log(S)]/Log(2), where, H = cell number harvested and S = cell number seeded.

**Assessment of trilineage differentiation potential of MSCs**

Passage 3 MSCs were harvested when they reached 80% confluence and were induced to undergo adipogenic, osteogenic and chondrogenic differentiation under specific culture conditions as follows.

**Adipogenic differentiation**

MSCs were seeded in a well of a sterile 6-well plate at a density of 1x10^5^ cells/ml suspended basal medium. When cells reached confluence, medium was substituted with StemMACS^TM^ AdipoDiff medium (Miltenyibiotec, Germany) and cells were maintained in culture for 21 days with medium replacement every 3 days. On day 21 cells were cells stained with 0.3% Oil-Red-O (Sigma) and histochemically assessed for lipid droplet detection.

**Osteogenic differentiation**

Cells were seeded in a well of a 6 well plate at a density of 3x10^4^ cells/well in StemMACS^TM^ OsteoDiff medium (Miltenyibiotec, Germany). After 24h, medium was completely replaced by fresh differentiation medium and cells were maintained in culture for 21 days with medium replacement twice a week. On day 21, cells were evaluated by alkaline phosphatase detection (Sigma) and by the von Kossa method for mineralisation assessment.

**Chondrogenic differentiation**

For chondrogenic differentiation 2.5x10^5^ MSCs were re-suspended in basal medium in a 15 ml polypropylene conical tube and centrifuged for 5 minutes at 150g, RT. The medium was completely removed and cells were re-suspended with pre-warmed StemMACS^TM^ ChondroDiff medium (Miltenyibiotec, Germany). Cells were centrifuged and incubated in a 37 ºC and 5% CO_2_ incubator as a pellet for 24 days with medium replacement every 2 to 3 days. On day 24, the cell pellet was fixed and sectioned into 5µm thin slices. The slides were then stained with 0.1 % Alcian Blue (Sigma) solution and microscopically assessed for proteoglycan.

**Supplementary Table S1: Surface protein screening of paired MSC-PLT and MSC-FCS samples.** Tables showing the antibody layout of plates 1-4, and the percentage of positive cells from plate 1, plate 2, plate 3 and plate 4 for MSC-FCS and MSC-PLT, as acquired using the MACSquantify analysis software. The surface proteins that exhibited > 5.5% on the positive gate were chosen for further validation of these results using the same system (excel spreadsheet).

**Supplementary Table S2**: **Percentage of cells expressing the positively detected surface markers.** Average, SEM, 95% confidence interval and coefficient of variation for each specific marker are shown. This analysis identified two groups or markers: one that was highly expressed across all samples in both MSC-PLT and MSC-FCS, i.e. exhibited a percentage of positive cells >70% and a MSC-PLT/MSC-FCS ratio of <1.5, and a second group of markers which show variable expression between samples and between MSC-PLT and MSC-FCS, which showed a percentage of positive cells >5.5% and a MSC-PLT/MSC-FCS ratio of >1.5. The markers showing significant differential expression between MSC-PLT and MSC-FCS show p-values <0.05.

**Supplementary Table S3: Summary of signal intensity values estimated by assessment of raw fluorescence intensity and calculation of stain index**. Results show the raw fluorescence intensity, herein estimated as Median fluorescence intensity of the percentage of positive population for each specific marker and the stain index values for each marker was calculated according to the formula: (MFIpos – MFIneg)/ 2xSDneg, where MFIpos = median fluorescence intensity of the % positive cells for each marker; MFIneg = median fluorescence intensity of the negative population, i.e., corresponding isotype control for each analysed marker and SDneg = standard deviation of the median fluorescence intensity of the negative population.

**Supplementary Table S4: Summary table showing networks associated with the overexpressed markers on MSC-PLT.** The enriched surface proteins were uploaded onto IPA software and analysed for their association with specific functions. The software predicted the involvement of these markers in three distinct networks associated with inflammatory responses, carbohydrate metabolism and cellular movement. Information about the score, focus molecules and the molecules in the network is presented.

| **Networks** | **Score** | **Focus**  **Molecules** | **Molecules in Network** |
| --- | --- | --- | --- |
| Network 1:  Inflammatory response | 28 | 11 | **ADGRE5**, Akt, **ALPL**, BPI, **CD40**, Cg, **CSPG4**, **DPP4**, ERK, ERK1/2, Histone h3, **HLA-E**, HTR4, **ICAM1**, IFNAR, IgG, IgG1, IL12(complex), Integrin, Integrin alpha 4 beta 1, **ITGA4**, JNK, NFKb complex, **NOTCH2**, P2RY4, P2RY6, P38 MAPK, **PECAM1**, PI3K (complex), RNA polymerase II, RNF40, SDC4, SFK, SLC52A1, **VCAM1** |
|
|
|
|
| Network 2:  Carbohydrate metabolism | 9 | 4 | ACP1, ADAM28, **ADGRE2**, **ADGRE5**, advanced glycation end-products, Akt, **ALPL**, **CDCP1**, Cg, CNR2, CYSLTR1, DUSP16, ERL, Focal adhesion kinase, FPR1, GAST, Gpcr, GPR4, Histone H3, HTR4, IKK (complex), Integrin, Integrin alpha 3 beta 1, MAPK, NFKb complex, P2RY1, P2RY4, P2RY6, P13K (complex), RNA polimerase II, S1PR2, SLC52A1, THBS2, VEGF |
|
|
|
|
|
| Network 3:  Cellular movement | 4 | 2 | ALP, ARAP1, ARFGEF2, BIRC2, CCL11, **CD40**, CYTH1, GHRH, GNB2L1, GPS2, GTPase, IPO5, IQGAP1, ITGA6, MADD, mevalonic acid, MLLT4, NISCH, **PECAM1**, PIN1, PLXNB2, PRKG1, RALGDS, RAP1GAP, RASGRF1, RCC1, RICTOR, S1PR1, SEMA4D, THY1, TRIO, WNT5A |
|
|
|
|

**Abbreviations:** ADGRE5: adhesion G protein-coupled receptor E5; AKT: (PKB) protein kinase B; ALPL: alkaline phosphatase liver/bone/kidney; BPI: bactericidal/permeability-increasing protein; CD40: cluster of differentiation 40; CSPG4: chondroitin sulphate proteoglycan 4; DPP4: Dipeptidyl peptidase-4; ERK: extracellular signal-regulated kinases; HLA-E: human leukocyte antigen E; HTR4: 5-hydroxytryptamine receptor 4; ICAM1: intercellular adhesion molecule 1; IFNAR: interferon alpha/beta receptor; IgG: immunoglobulin G; IL12: interleukin 12; ITGA4: integrin alpha 4; JNK: c-Jun N-terminal kinases; ACP1: acid phosphatase 1; ADAM28: ADAM metallopeptidase domain 28; CDCP1: CUB domain containing protein 1; Cg: class C-G-protein coupled receptors: CNR2: cannabinoid receptor 2; CYSLTR1: cysteinyl leukotriene receptor 1; DUSP16: dual specificity phosphatase 16; ERL: endoplasmic reticulum lipid; FPR1: formyl peptide receptor 1; GAST: gastrin; Gpcr: G protein-coupled receptor; GPR4: G protein-coupled receptor 4; IKK: IkB kinase; MAPK: mitogen-activated kinases; NFkB: nuclear factor k beta; P2RY(number): purinergic receptor P2Y, G-protein coupled (number); P13K: phosphpoinositide 3-kinase; S1PR2: sphingosine-1-phosphate receptor 2; SLC52A1: solute carrier family 52 member 1; THBS2: thrombospondin 2; VEGF: vascular endothelial growth factor; ARAP1: arfGAP with RhoGAP domain, ankyrin repeat and PH domain 1; ARFGEF2: adenosine phosphate-ribosylation factor guanine nucleotide-exchange factor 2; BIRC2: baculoviral IAP repeat containing 2; CCL11: chemokine ligand 11; CYTH1: cytohesin 1; GHRH: growth hormone releasing hormone; GNB2L1: guanine nucleotide binding protein, beta polypeptide 1-like 1; GPS2: G protein pathway suppressor 2; GTPase: guanine triphosphatase; IPO5: importin 5; IQGAP1: IQ motif containing GTPase activating protein 1; ITGA6: integrin alpha 6; MADD: MAP-kinase activating death domain; MLLT4: myeloid/lymphoid leukemia translocated to 4; NISCH: nischarin; PIN1: peptidylpropyl cis/trans isomerase NIMA-interacting 1; PLXNB2: plexin B2; PRKG1: protein kinase, CGMP-dependent type 1; RALGDS: ral guanine nucleotide dissociation stimulator; RAP1GAP: RAP1 GTPase activating protein; RCC1: regulator of chromosome condensation 1; RICTOR: RPTOR independent companion of MTOR, complex 2; S1PR1: sphingosine-1-phosphate receptor 1; SEMA4D: sema domain, immunoglobulin domain, transmembrane domain and short cytoplasmic domain; THY1: Thy-1 cell surface antigen; TRIO: trio rho guanine nucleotide exchange factor; WNT5A: wingless-type MMTC integration site family, member 5A; RNF40: ring finger protein 40.

**Supplementary Table S5: Antibodies used for Lyoplate analysis.** Antibodies with respective plate ID, clone, isotype control and supplier are described.

**Supplementary Table S6: Antibody and data acquisition information for multiparameter flow cytometry.**

| Antigen | Fluorochrome | Clone | Isotype | Detection Channel |
| --- | --- | --- | --- | --- |
| CD318 | FITC | REA194 | REA hu IgG1 | 488nm 530-30 |
| CD54 | PE-Vio770 | REA266 | REA hu IgG1 | 561nm 710-50 |
| CD26 | PE | FR10-11G9 | mIgG1 | 561nm 585-15 |
| CD312 | APC | REA302 | REA hu IgG1 | 640nm 670-30 |
| MSCA-1 | Streptavidin BUV395 | W8B2 | mIgG1 | 355nm 379-228 |
| CD106 | APC-Vio770 | REA269 | REA hu IgG1 | 640nm 780/60 |

| CD49d | APC | MZ18-24A9 | mouse IgG2b | 640nm 670-30 |
| --- | --- | --- | --- | --- |

**Supplementary Figure S1: qRT-PCR analysis of MSCA-1, CD26, CD318**

**
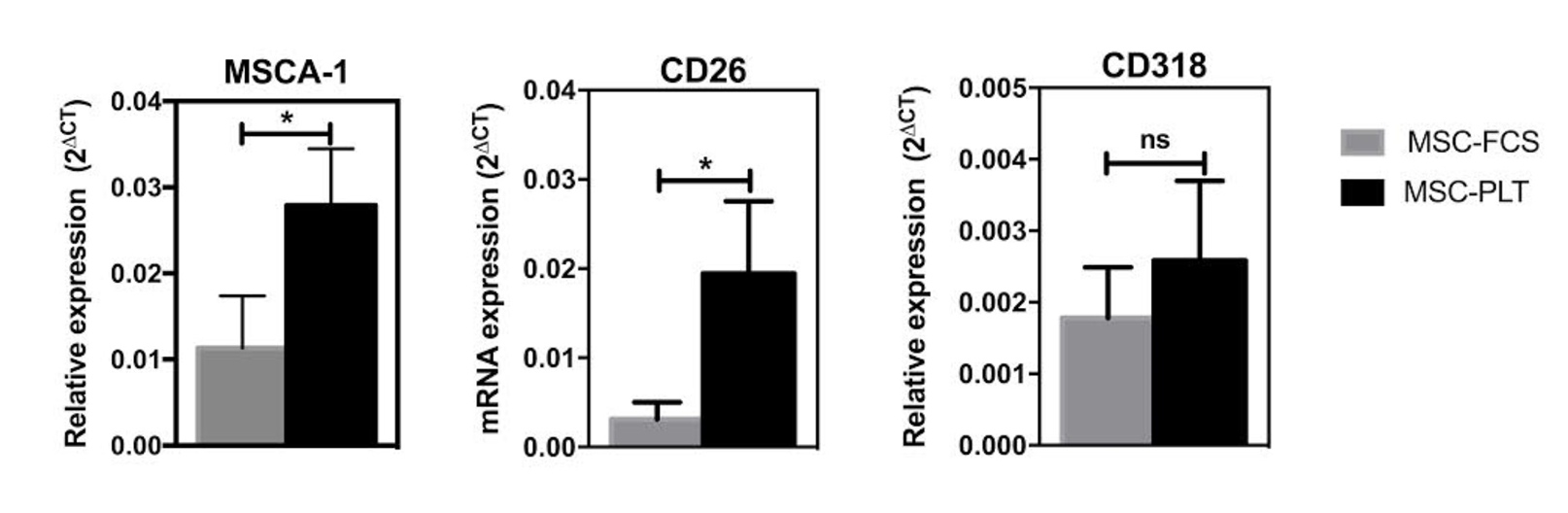
**

The expression of *ALPL* (MSCA-1), *DPP4 (*CD26)*,* and *CDCP1 (*CD318) was examined in an independent cohort of paired MSC samples (n=3). The expression level shown was relative to *GAPDH* endogenous control. Error bars represent mean ± SEM and * p-value<0.05.
